# Canonical and Noncanonical Functions of the BH3 Domain Protein Bid in Apoptosis, Oncogenesis, Cancer Therapeutics, and Aging

**DOI:** 10.3390/cancers16122199

**Published:** 2024-06-12

**Authors:** Yetunde Makinwa, Yibo Luo, Phillip R. Musich, Yue Zou

**Affiliations:** 1 Department of Cell and Cancer Biology, University of Toledo College of Medicine and Life Sciences, Toledo, OH 43614, USA; yetunde.makinwa@utoledo.edu (Y.M.); yibo.luo@utoledo.edu (Y.L.); 2 Department of Biomedical Sciences, Quillen College of Medicine, East Tennessee State University, Johnson City, TN 37614, USA; musichp@mail.etsu.edu

**Keywords:** cancer, apoptosis, mitochondria, BH3 domain, Bid, ATR, oncogenesis, chemoresistance, senescence, cancer therapy, LB-100

## Abstract

**Simple Summary:**

Cancer remains a debilitating disease and a worldwide health burden, and their treatment has been a forefront interest for researchers and clinicians for centuries. The search for novel approaches to selectively target certain proteins in just cancer cells, while sparing normal, healthy cells, has been the holy grail to limit side effects of treatments. This review aims to assess the potential of tBid, a pro-apoptotic mitochondrial protein found in normal and healthy cells, as a target for treating cancers. Cancer cells with tBid accumulation at mitochondria are “primed” for apoptosis, and, surprisingly, may be resistant to apoptosis owing to the association of tBid with antiapoptotic proteins. These cancer cells are more vulnerable to drugs that target these associations, with the possibility of limiting the use of chemo- or other cancer therapies.

**Abstract:**

Effective cancer therapy with limited adverse effects is a major challenge in the medical field. This is especially complicated by the development of acquired chemoresistance. Understanding the mechanisms that underlie these processes remains a major effort in cancer research. In this review, we focus on the dual role that Bid protein plays in apoptotic cell death via the mitochondrial pathway, in oncogenesis and in cancer therapeutics. The BH3 domain in Bid and the anti-apoptotic mitochondrial proteins (Bcl-2, Bcl-XL, mitochondrial ATR) it associates with at the outer mitochondrial membrane provides us with a viable target in cancer therapy. We will discuss the roles of Bid, mitochondrial ATR, and other anti-apoptotic proteins in intrinsic apoptosis, exploring how their interaction sustains cellular viability despite the initiation of upstream death signals. The unexpected upregulation of this Bid protein in cancer cells can also be instrumental in explaining the mechanisms behind acquired chemoresistance. The stable protein associations at the mitochondria between tBid and anti-apoptotic mitochondrial ATR play a crucial role in maintaining the viability of cancer cells, suggesting a novel mechanism to induce cancer cell apoptosis by freeing tBid from the ATR associations at mitochondria.

## 1. Introduction

Homeostatic imbalance within cells can lead to cancer, and programmed cell death via apoptosis serves as a significant, evolutionarily conserved failsafe mechanism to eliminate these aberrant cells [1,2]. However, failure of apoptosis can result from many causes, and this failure is recognized as one of the major hallmarks in cancer [3,4]. Of the many ways by which a cell tries to prevent oncogenesis, apoptosis is especially important as it might serve as a last-ditch effort the cell falls on if other interventions (oncogene inactivation, tumor suppression, cell cycle checkpoints, etc.) fail. It is also the downstream process by which upstream death-inducing processes (such as DNA damage, oncogene activation, failure of tumor suppression) are funneled through. The importance of the apoptotic process is underscored by the numerous cancer therapies targeted at regulators of the apoptotic pathway [5,6].

**Figure 1:**
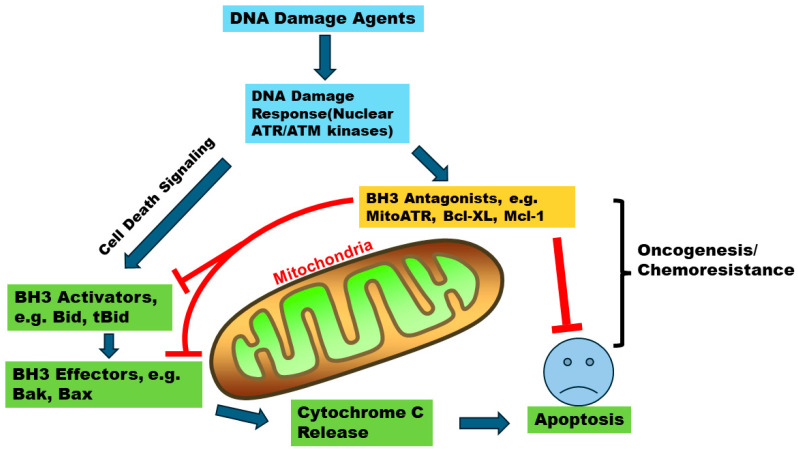
A graphical illustration of the roles of Bid/tBid and mitochondrial ATR in apoptosis and oncogenesis. MitoATR and other anti-apoptotic Bcl-2 proteins (e.g., Bcl-XL) bind and sequester tBid, at the OMM, so that it is unavailable to activate Bax/Bak for apoptosis. These proteins can also be overexpressed in oncogenesis and in the development of chemoresistance.

There are many cell regulators of apoptosis, and the mitochondrion serves as a central organelle since it is associated with the BH3 family of proteins including both pro- and anti-apoptotic proteins, who play key roles in the intrinsic apoptotic pathway. The mitochondrial outer membrane is where these proteins converge to act (Figure 1). These proteins govern cell fate by regulating mitochondrial outer membrane permeabilization (MOMP) and the release of cytochrome C; the latter activates the caspase cascade [7], and ultimately leads to cell death. The BH3 family of proteins and the roles of its pro- and anti-apoptotic members are well defined. Members of this family share variants of a single sequence motif commonly known as the BH3 domain. Among all the BH3 family proteins, the ratio of Bax/Bcl-2 is essential to determine the cell fate in response to apoptosis stimuli. The increased ratio leads cells to death and decreases tumor occurrence [8]. Colon cancer patients above 50 have decreased Bax/Bcl-2 ratio, suggesting that it can be regarded as a prognosis marker [9]. Increasing the ratio of Bax/Bcl-2 is associated with better prognosis and survival in patients with leukemia [10]. Bid (BH3-interacting domain death agonist) protein is a member of the BH3 family and performs its pro-apoptotic function via its truncated form, tBid, which is found as highly expressed in 6% of solid tumors [11]. A study has found that the well-known *Ataxia–Telangiectasia* and Rad-3-related (ATR) kinase is also a new BH3 domain-containing protein [12]. After DNA damage, while nuclear ATR coordinates DNA damage responses and repair processes, cytoplasmic ATR translocates to the mitochondria. Here, the exposed BH3 domain of mitochondrial ATR (mitoATR) enables it to act as an anti-apoptotic protein by interacting with tBID [12], therefore inhibiting its activity. Consequently, tBID emerges as a pivotal determinant that dictates the cell fate between apoptosis and carcinogenesis. In this review, we will focus on a discussion of how Bid protein plays an important role in the convergence of these two alternative cell fates.

## 2. Bid’s Role in Mitochondrial Apoptosis

The BH3 family of proteins belongs to the B cell lymphoma 2 (Bcl-2) super-family of proteins. The Bcl-2 family regulates the permeability of the outer mitochondrial membrane. Generally, the Bcl-2 family members are grouped as either pro- or anti-apoptotic. They also can be grouped based on the Bcl-2 homology (BH) domains they possess: BH-1, -2, -3, and -4. Although not all Bcl-2 family proteins contain multiple BH domains, they all contain the BH3 domain. Bid is a member of the BH3-only protein family (so named because they only have the BH3 domain). This domain is important as it is exposed when Bid is proteolytically activated into tBid, which binds with high affinity to other Bcl-2-like proteins and triggers apoptosis [13,14,15]. Several in vitro and in vivo [16] studies confirmed its role in initiating apoptosis.

The cleavage of Bid into tBid can occur via caspases, especially caspase 8, or by granzyme B, calpains, and cathepsins [17,18]. tBid is the C-terminal truncate of Bid and translocalizes, after truncation, to the mitochondrial outer membrane for apoptotic activation by promoting the oligomerization of the pro-apoptotic Bax and Bak proteins [19]. Bax and Bak homo-oligomers are then inserted into the outer mitochondrial membrane (OMM) to elicit MOMP (mitochondrial outer membrane permeabilization) and leakage of mitochondrial proteins such as cytochrome C and apoptosis-inducing factor (AIF) into the cytosol [18]. Once in the cytosol, cytochrome C activates caspases through binding to apoptotic protease activating factor 1 (Apaf-1), leading to cell death [20,21,22,23,24]. Bid is the central link between extrinsic and intrinsic apoptosis. Both extrinsic signals, like Fas signaling, and intrinsic cellular stress can trigger caspase 8 activation, leading to the cleavage of Bid and the generation of tBid, as a starting point of the apoptosis process.

A noncanonical activation of Bid has also been described in mitochondrial apoptosis during mitotic arrest. Here, the phosphorylation of Bid on serine 66 primes the cells to undergo intrinsic mitochondrial apoptosis if the mitotic exit is delayed [25]. This study used colon carcinoma cell lines as the model. Further investigation is required to determine whether the noncanonical activation mechanism applies to other types of cancer cells. Notably, a lung cancer study has shown that Bid overexpression during mitosis confers increased resistance to apoptosis induced by mitotic kinase inhibitors [11], suggesting that the Bid level is worthy of consideration in clinical treatment. In addition, tBid can induce MOMP by itself, followed by release of cytochrome C and mitochondrial DNA, and thus, apoptosis independent of BAX and BAK [26].

## 3. Cytoplasmic ATR’s Role in Mitochondrial Apoptosis

Once tBid has reached the mitochondria, apoptosis will occur unless halted by other interactions. The BH3 domain of tBid can be bound by other BH3 domain-containing proteins at the OMM, e.g., tBid itself to form homo-oligomers [27,28] or the Bax/Bak molecules they have activated, or, alternatively, tBid can be sequestered by anti-apoptotic proteins such as Bcl-XL to inhibit the permeabilization of the OMM [29,30]. A recently identified BH3-containing protein that can bind and sequester tBid upon DNA damage is the cytoplasmic/mitochondrial form of ATR [12]. This is particularly important because DNA damage is believed to be the major cause of DNA mutations, thus cancer, and the presence of an anti-apoptotic protein such as mitochondrial ATR (mitoATR), can inhibit the activation of BAX and subsequent apoptosis, despite the presence of death signals [12,31].

Traditionally, ATR is well known for its kinase function in the nucleus. It interacts with several other nuclear proteins in its role in the cell’s response to DNA damage and repair [32,33,34,35,36,37]. However, following DNA damage, cytoplasmic ATR undergoes prolyl isomerization at the ATR^S428P429^ motif to transform from *trans*-ATR to *cis*-ATR with a now-exposed BH3 domain at its N-terminus. When ATR is phosphorylated at S428, peptidyl-prolyl *cis*-*trans* isomerase Pin1 catalyzes the ATR transform from *cis* to *trans* at the S428-P429 motif. In response to DNA damage, Pin1 activity is inhibited by T71 phosphorylation. Additionally, PP2A dephosphorylates ATR at S428. These two pathways collectively maintain ATR in its *cis* isoform. This *cis*-ATR translocalizes to the OMM (so-called mitoATR) to bind and sequester tBid (Figure 1). This sequestered tBid is now unavailable for inducing Bax/Bak oligomerization, thus arresting cytochrome C release and the pro-apoptotic process [12,31,38].

It has been reported that the expression of cytoplasmic ATR increased in ovarian cancer cells upon the acquisition of chemoresistance [39]. This cytoplasmic ATR can translocate to the OMM of the mitochondria following death signals [12]. Its exposed BH3 domain then allows ATR to make key associations with other BH3 domain proteins like tBid at the mitochondria. This has important implications in determining cell fate—apoptosis, continued immortality, or treatment resistance [40].

## 4. tBid at the Mitochondrion Plays a Role in the Dueling and Converging Fates of Cells (Apoptosis vs. Oncogenesis)

In healthy cells, the mitochondria act as a nexus for processing dueling signals—pro- and anti-apoptotic signals from endogenous or exogenous sources. When a healthy cell sustains irreparable damage from various sources, it is marked for death. The conversion of Bid to its activated form, tBid, at the mitochondria ensues, leading to compromised mitochondrial outer membrane permeabilization (MOMP). Once this occurs, the cell is at the point of no return along this pathway of apoptosis, irreversibly committed to die. However, cancer cells have evolved mechanisms to evade apoptotic cell death, enabling them to achieve immortality despite encountering death signals triggered by irreparable damage from mutations or genotoxic stress. Any perturbations in BH3 family proteins may have important implications in the development of cancers [41,42].

Numerous studies have documented an overexpression of Bid protein in various cancers, including leukemia and liver cancer [42,43,44,45]. Mutations on Bid are the main genetic alterations that lead to the overexpression of Bid. Through these mutations, Bid protein may increase its half-life [46], and fail in inducing cytochrome C release and Bcl-2 or Bax interaction [47]. All these genetic changes accumulate Bid within cells. However, several studies found a positive relation between apoptosis activity and a poor prognosis. Apoptosis also correlates with a higher mitotic index in cancer cells, indicating an essential link between apoptosis and cancer cell proliferation. As mentioned above, Bid phosphorylation at S66 is particular during mitosis and plays a key role in mitotic arrest-induced apoptosis. All these evidences show that the genetic alteration and regulation of Bid are key to cancer cell survival and prognosis.

It is worth highlighting that it is the tBid, the truncated form of Bid, that serves as the trigger for apoptosis. This has led to the development of treatment strategies aimed at increasing cellular levels of tBid [48,49,50,51,52,53,54,55,56,57]. The aim is to achieve a predominance of the pro-apoptotic tBid, which would then prompt cancer cells to undergo apoptosis via the activation of Bax/Bak. Thus, Bid protein potentially assumes a dual role in both oncogenesis and cancer treatment through its conversion to tBid. A recent in vivo study found that Bid also functions parallel to Bax/Bak in embryonic development-associated apoptosis rather than only acting upstream of them. Moreover, full-length Bid, not the tBid mentioned above, can also induce a small portion of cytochrome C release by forming polymers by itself [58].

The battle between pro- and anti-apoptotic forces dictates whether cells succumb to death or attain immortality [59]. Although chemotherapy stands as a cornerstone in cancer treatment, the development of chemoresistance poses a significant challenge to its efficacy. BH3-containing proteins emerge as pivotal players in the development of chemoresistance. For instance, acquired chemoresistance in pancreatic cancer cells and lymphoma is linked to the overexpression of Bid and other Bcl-2 family proteins [60,61]. Another mechanism impeding the success of chemotherapy involves the occupation of the BH3 domain on pro-apoptotic factors, hindering their activity. For instance, mitoATR interacts with tBid to curtail its activity, thwarting the apoptotic process. Another Bid binding partner, PACS2, which is essential for ER and mitochondrial communication, can interact with and help Bid translocate onto mitochondria to execute its pro-apoptotic role. This translocation is in response to the apoptosis signals [62].

## 5. Bid Oncogene Addiction and Bid/tBid-Mediated Mitochondrial Priming of Cancer Cells

As a result of the increased expression of basal Bid/tBid protein following accumulated DNA damage [42], certain tumor cells exhibit a peculiar dependence on this pro-apoptotic factor [63,64,65,66,67]. Describing these cells as “addicted” to Bid underscores their reliance on its presence for maintaining viability and highlights a unique vulnerability that can be exploited therapeutically. Indeed, such cells become markedly more susceptible to treatments aimed at modulating the cellular abundance or functionality of Bid. This enhanced sensitivity can be attributed to the state of being “primed” by the BH3 domain of activated Bid (tBid), wherein these cells find themselves teetering at the brink of the apoptotic precipice and are near the end of the apoptotic signaling (in the execution phase of apoptosis) [64,68,69]. Consequently, their mitochondrial threshold for being fully committed to cell death is much lower, a condition termed “primed to die”. These primed cells are more vulnerable to the synthetic lethality approach of killing with a single agent targeted at a specific BH3-only protein (tBid at the mitochondria) to tip the cell over the threshold needed for a rapid apoptotic death [70,71,72,73,74].

The intriguing aspect of these “primed for death” cells lies in their continual reliance on the presence of anti-apoptotic proteins at the mitochondria to forestall the inevitable transition into apoptosis [12,75]. Herein lies the crucial role of mitoATR, which functions as a staunch guardian against the final activation of apoptosis. It has been shown that there is a co-overexpression of mitochondrial ATR together with tBid in cancer cells [12,31]. Remarkably, these two entities form a stable complex at the outer mitochondrial membrane (OMM), effectively obstructing the initiation of the apoptotic cascade at the “primed intermediate stage” [9,27]. Agents capable of reducing mitoATR levels, or disrupting the mitoATR-tBid association, may represent promising avenues for intervention. Such interventions free tBid from its sequestration, allowing it to fully execute the apoptotic program [31,67].

In essence, the intricate interplay between Bid, mitoATR, and other regulatory proteins underscores the delicate balance between cell survival and programmed cell death. Exploiting this balance through targeted interventions holds great promise for developing novel therapeutic strategies against cancer cells primed for death.

## 6. Importance of BH3 Family Protein, Bid, in Cancer Treatment and Development of Chemoresistance

The major roles played by the BH3 family of proteins in mitochondrial apoptosis and the differential expression of the members of this family in cancers have made them an obvious target. Thus, the BH3 family proteins have been proposed as molecular targets in treatment of cancers, with BH3 inhibitors [76,77,78] and mimetics [79,80,81] currently being tested in several clinical trials, especially in treatments targeted at overcoming chemoresistance [82]. However, there are several limitations and challenges in directly targeting these proteins [81,83,84].

With high levels of cellular stress, apoptotic cell death may occur quickly. However, with low levels of cellular stress, cells can accumulate DNA damage (such as with a normal aging process). Although this might not eventually lead to apoptosis, there can be an accumulation of tBid and anti-apoptotic BH3 proteins at the mitochondria, leading to a “primed” state and the cell stays alive, where further low-level stress can tip the cell into eventual apoptosis. While healthy cells have a well-regulated balance between the expression of pro- and anti-apoptotic proteins, we and others have observed an overexpression of the Bid/tBid protein in cancer cells at the diagnosis or after the development of acquired chemoresistance. The development of acquired treatment resistance in cancers may be attributed to the cell’s evasion of apoptosis. One of the mechanisms described has been the overexpression of anti-apoptotic proteins with BH3 domains, such as tBid [42], BCL-2 [74,85,86], BCL-XL [17], and mitoATR [12], or inhibitors such as XIAP, clAPs, and surviving BIRC5 [87,88,89]. Despite the abundance of the pro-apoptotic Bid/tBid, the cells resist apoptosis. This may be attributed to the presence of anti-apoptotic interacting partners of tBid at the mitochondria. MitoATR is one such partner that acts as a brake to the already advanced apoptotic status in these “primed” cells. There are also other possible explanations: (a)—Inducing anti-apoptotic protein expression at both the transcription and translation levels [90]. (b)—Altering protein functions. There are several possible mechanisms to explain the protein function change from pro-apoptotic to non-functional. One is that pro-apoptotic proteins only function at a specific time depending on the intensity of death signaling. Another possibility is that the caspase cascade proteins can induce the expression of other genes. SMAC, a mitochondrial protein, which is a pro-apoptotic protein, is widely expressed in many kinds of cancer cells. Besides its pro-apoptotic function, it is also involved in gene expression (such as p53), lipid homeostasis, and embryonic development [91]. (c)—Pro-apoptotic protein degradation by proteasomes. How do these mechanisms differing across various cancer types remain vague due to the complexity of these anti-apoptotic mechanisms identified in numerous types of cancer cells? Importantly, these mechanisms are not mutually exclusive and cancer cells may employ one or multiple mechanisms to evade apoptosis [92].

Though Bid is a long-lived protein, the half-life of tBid is rather short. A 26S proteosome is responsible for tBid degradation and its binding to either Bax or Bcl-2 has no effect on its degradation [46]. Human and mouse models both demonstrated that a 26S proteosome is post-transcriptionally highly expressed in cancer cells, which may degrade tBid to stop the apoptosis pathway [93].

**Figure 2:**
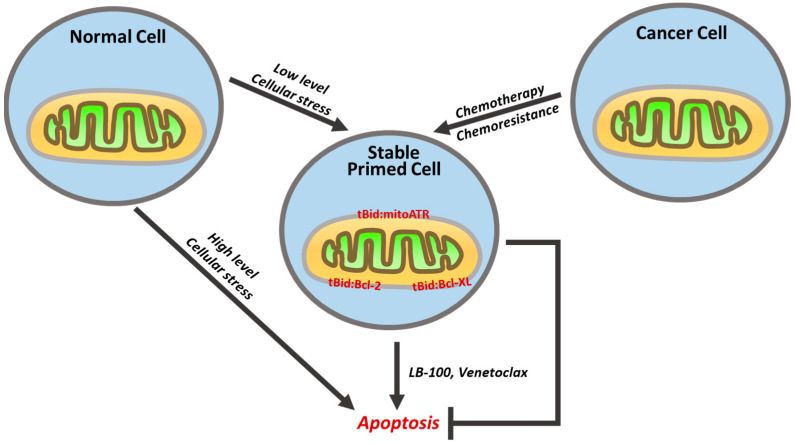
A graphical illustration of the stable associations between tBid and anti-apoptotic proteins like mitoATR, Bcl-2, and Bcl-XL at the OMM. Low levels of cellular stress in healthy cells (e.g., DNA damage accumulation with aging) or chemotherapy in cancer cells might not kill the cells but can create stable states of primed cells with tBid-protein associations at the OMM. Perturbations in these tBid-protein associations (e.g., LB-100 reduces mitoATR production, venetoclax binds the BH3 domains of Bcl-2 and Bcl-XL) free tBid to activate Bax/Bak. The stable primed cells are primed for killing with single-agent drugs.

It would be an interesting effort to develop molecule inhibitors to disrupt the interaction of tBid with its antiapoptotic interacting partner(s) in these “primed” cells. This action would reverse the sequestration of tBid, thereby rendering it available to serve as the ‘final nudge’ required by the cells to complete the mitochondrial apoptotic pathway. [75,94]. In cancer cells with an overabundance of tBid bound by Bcl-2, the small molecule inhibitor (e.g., venetoclax) can bind to the exposed BH3 domain of Bcl-2 in a competitive manner, and free tBid for Bax/Bak activation, making only these cancer cells highly susceptible to its action, sparing healthy cells. The same principle applies to cancer cells with an overabundance of mitoATR, involving the use of a small molecule inhibitor of PP2A (LB-100) that reduces the availability of mitoATR and allows tBid to be free. For chemoresistant cancer cells with high tBid expression, as the apoptotic signaling is already highly primed by tBid, only a low dose of a drug that targets the tBid anti-apoptotic protein associations at the mitochondria could be adequate to trigger the apoptotic signal (Figure 2). The use of a single-agent drug may give an added advantage of bypassing the numerous side effects attributed to conventional treatments such as chemo- and radiation or immune therapies.

## 7. Bid/tBid as a Potential Factor in Aging and Aging-Related Disorders

As discussed earlier, a high level of cellular stress can trigger cell death, whereas low levels of cellular stress led to the accumulation of DNA damage without immediate cell demise. Aging exemplifies such a phenomenon. With aging, the efficiency of DNA damage repair declines, resulting in the accumulation of DNA damage [95]. Consequently, DNA damage accelerates the aging process [80]. In the context of cellular senescence, the overexpression of p16 and p21 halts the cell cycle in senescent cells, rendering the elimination of p16-positive cells a therapeutic approach to slow down or even reverse aging and aging-related disorders [96,97,98].

Besides targeting p16, BH3 domain proteins have also drawn wide attention recently. An RNAseq analysis reveals a significant increase in pro-apoptotic factors such as PUMA, Bax, Bid, and BIM, alongside the downregulation of anti-apoptotic factors like Bcl-2 [97]. This scenario resembles that observed in chemoresistant cancer cells, where the apoptosis threshold is lowered, yet cell death is not induced. Interestingly, senescent cells also exhibit resistance to apoptosis. Several studies have highlighted the role of an inefficient p53 network, inactivation of the NF-kappa B pathway, and epigenetic dysregulation in contributing to this resistance [99]. Recent findings in cancer cells, indicating that mitoATR interacts with tBid to inhibit its activity [12], may also apply to cellular senescence and aging. It is of interest to determine whether cellular senescence exhibits enhanced levels of mitoATR and tBid interaction at the mitochondria due to DNA damage accumulation. Furthermore, any intervention capable of altering this interaction may prompt apoptosis in senescent cells. This underscores a possible novel mechanism underlying apoptotic resistance in senescent cells.

In addition to directly targeting the interaction between mitoATR and tBid, it is also intriguing to investigate the regulation of Bid/tBid and ATR following DNA damage. ATR, as a pivotal coordinator of the DNA damage response (DDR) pathways, primarily operates in the nucleus to phosphorylate various substrates in DDR such as Chk1/2 and p53. Interestingly, Bid has been observed to accumulate in the nucleus apart from its cytoplasmic localization [100], similar to other BH3 domain-containing proteins in cells without prolonged passaging. What functions do they fulfill in the nucleus? How do they translocate to the cytoplasm? And with what molecular partners do these BH3 domain-containing proteins interact in response to DNA damage? Unraveling the answers to these questions may help our understanding of the intricate interplay among these proteins. Moreover, shedding light on these mechanisms is invaluable for drug development aimed at overcoming the blockade of apoptosis in senescent and cancer cells.

## 8. Bid/tBid and mitoATR Translocation and Regulation

Nuclear Bid has been identified to exhibit an S-phase checkpoint function by upregulating p21 and p27, independent of p53, when low levels of DNA damage are present [44]. However, when the DNA damage level surpasses a certain threshold, Bid shuttles to the cytoplasm to be cleaved by caspase 8, generating truncated tBid, which subsequently translocates to the mitochondrial outer membrane to initiate apoptosis. The nuclear export receptor CRM1 and p53 facilitate the transport of Bid out of the nucleus. In the presence of DNA damage, Bid mRNA is increased in a p53-dependent manner [101], and nuclear Bid is phosphorylated by ATM to execute its S-phase arrest role [102]. In addition to its S-phase arrest role, Bid accumulates in nuclei more than in the cytoplasm of epithelial cells from metastatic liver tumors rather than from normal liver tumors [103].

To date, there is no direct evidence supporting the role of CRM1 and p53 in facilitating the translocation of ATR out of the nucleus. However, a structural analysis has revealed that p53 interacts with the BH3 binding pocket of the pro-apoptotic protein BCL-2 to inhibit the latter’s activity [104]. The studies mentioned above collectively suggest that CRM1 and p53 may recognize BH3 domain-containing proteins by binding to the BH3 pocket, thereby influencing their localization and functions. Therefore, investigating the mechanisms underlying the nuclear–cytoplasmic shuttling and regulation of Bid, ATR, and other BH3 domain-containing proteins in response to DNA damage is crucial for future research endeavors.

## 9. Targeting mitoATR-tBid Interaction as a Strategy for Cancer Therapeutics

Given the pivotal role of Bid/tBid as a linker between apoptotic cell death and oncogenesis, cancer treatments can take advantage of this role of Bid/tBid to develop better and more efficient cancer treatments, but with fewer off-target effects. This is because cancer cells, which have a higher expression of anti-apoptotic mitoATR and pro-apoptotic tBid, are at a precarious homeostatic balance at the cell OMM. This creates a situation of oncogenic addiction to both proteins and has been observed both in cancer cells at initial diagnoses/treatments and after the development of chemoresistance. Cellular dependence on these proteins for survival therefore makes them more vulnerable to being targeted and killed by tipping the balance off-kilter by destabilizing the stable association between mitoATR and tBid that is preventing the cell from tipping into apoptosis via the intrinsic pathway. More work remains to be done to further understand and characterize fully the mitoATR-tBid interactions at the OMM; hence we propose that targeting of the tBid binding could be achieved using drugs currently available, such as PP2A inhibitors, LB-100 that makes mitoATR unavailable. Several clinical trials aimed to treat ovary cancer and small cell lung cancer have embraced the combined application of DNA damage drugs and LB-100 due to the recognition of PP2A as a key factor in tumorigenesis. Given that mitoATR impedes tBid post DNA damage, targeting PP2A specifically in tumors following the development of resistance to DNA damage drug therapy holds considerable promise. The end goal is the destabilization of the mitoATR/tBid complex at the OMM in these “primed” cells, freeing tBid to perform its pro-apoptotic role and tipping the cell into the death pathway while sparing healthy, non-“primed” cells. This could have significant implications for achieving the ultimate goal of more targeted cancer treatments with minimal off-target effects, which currently contribute significantly to the morbidity and mortality associated with cancer therapy.

## 10. Conclusions

BH3 domain-containing proteins primarily participate in cell survival mechanisms. However, in cancer or senescent cells where DNA damage is persistent, these proteins undergo functional dysregulation in terms of their localization and expression, thereby engaging in “death signaling”. Among these proteins, Bid/tBid holds particular significance as it initiates the apoptosis cascade by modulating BAX/BAK oligomerization and subsequent cytochrome C release. In the scenarios featuring low levels of DNA damage, the activity of tBid is consistently silenced by its interaction, for instance, with mitoATR through the BH3 domain, leading to chemotherapy resistance in cancer and the accumulation of aging cells. The disruption of the mitoATR/tBid interaction, therefore, may restore apoptosis in chemoresistant cancer cells and senescent cells. It is noteworthy that this interaction may only occur under conditions of chronic DNA damage. Thus, by targeting mitoATR, apoptosis may be selectively induced in cancer or senescent cells without affecting healthy cells.

Future investigations may need to thoroughly unveil the molecular mechanisms underlying Bid’s cellular functions related to cancer. This includes the following: (1) Understanding how Bid is regulated at the transcription and translation levels, particularly its post-translational modifications following apoptosis stimuli, and its trafficking between the nucleus and cytoplasm. (2) Investigating the potential for detecting the phosphorylation status of ATR(S428) and the mitoATR-tBid PLA signal as a diagnosis and prognosis indicator of cancer. (3) Exploring the interaction between Bid and mitoATR, a key factor in mitochondrial DNA damage response. (4) Examining the regulation of PP2A activity on mitochondria within the context of DNA damage, with the goal of elucidating the specific pathways that regulate the interaction between ATR and tBid on mitochondria. (5) Developing strategies to disrupt the mitoATR-Bid interaction in chemoresistant tumors and aging cells, aiming to overcome resistance mechanisms and improve treatment efficacy.
